# Second language learning role-play: effects of patient and doctor roles on motivation and competence

**DOI:** 10.3389/fmed.2023.1163267

**Published:** 2023-06-14

**Authors:** Hao Yu, Anna Isahakyan, Jeroen J. G. van Merrienboer, S. Eleonore Köhler, Maryam Asoodar

**Affiliations:** ^1^School of Health Professions Education, Faculty of Health, Medicine and Life Sciences, Maastricht University, Maastricht, Netherlands; ^2^Department of Anatomy and Embryology, Maastricht University, Maastricht, Netherlands

**Keywords:** peer role-play, medical L2 learning, intrinsic motivation, feeling of relatedness, competence

## Abstract

**Objectives:**

Role-playing has motivated foreign language learners for decades. In doctor–patient medical consultation role-plays, the doctor role has always been considered an important learning opportunity, whilst the patient role remained obscured. Our study, therefore, had a dual focus. We first explored how intrinsic motivation changes medical second-language (L2) learning through the lens of self-determination theory. We subsequently examined if playing the role of the patient provides additional value to medical L2 learning.

**Methods:**

We performed a mixed-methods study using a one-group pretest–posttest design. Participants were 15 student volunteers learning medical Dutch through peer role-play in medical consultations. Students completed a questionnaire before and after the course that measured changes in their intrinsic motivation to experience stimulation (IMES), feeling of relatedness, and feeling of competence. We also measured students' competence through a peer-rated checklist and the final course grades. At the end of the course, the students participated in semi-structured interviews to discuss their experience acting as patients. The data were subjected to the Wilcoxon signed-rank test and a thematic analysis.

**Results:**

The pre- and post-questionnaires revealed that students' IMES as well as their feeling of relatedness increased. Their self-perceptions, feeling of competence, peer assessments, and final course grades demonstrated that students were competent in medical L2. Our thematic analysis led to the identification of five themes of the role-play exercise for medical L2 learning: (1) motivational experience, (2) supportive peer interaction, (3) setting up a role-play environment for medical L2 learning, (4) utilizing the patient role to benefit medical L2 learning, and (5) a novel patient perspective on the doctor's role.

**Discussion:**

Our study found that role-play, by enhancing students' intrinsic motivation, feeling of relatedness, and competence development, aids the medical L2 learning process. Interestingly, playing a patient role in medical consultation was also found to support this process. We welcome future controlled experiments to confirm the positive impact of playing the role of the patient in medical consultation.

## Introduction

Role-playing strategies describe students talking and acting in accordance with a certain character (1, 2). Such strategies are widely applied in communication and second-language (L2) learning (3, 4). The findings of Nestel and Tierney (5) indicate that role-playing can be a valuable tool for enhancing the communication skills of 1st-year undergraduate medical students. Similarly, Bagacean and Cousin (6) have utilized role-play to cultivate empathy and communication skills in medical students. Altun (7) affirms the efficacy of role-playing activities in fostering motivation among students to learn foreign language speaking skills and to develop their communication competence. In medical consultation peer role-play, two students assume the roles of the doctor and patient, respectively, in order to practice L2 communication in a medical environment. According to Ladousse (8), “role-play is one of an array of communicative techniques that enhance fluency in language students, foster classroom interaction, and boost motivation.” (p. 7). We argue that, also in a medical consultation context, role-play can have a similar potential to foster learning.

Although scholars and teachers see role-playing as a motivating way to enhance student learning and interaction (9, 10), there has been little in-depth research conducted on this topic by researchers. According to the self-determination theory (11), students who pursue joy and excitement in learning have a high intrinsic motivation to experience stimulation (IMES) (12, 13). This atmosphere of pleasure, excitement, and interactivity is the focal point of what role-playing offers for language teaching in the classroom. Despite this fact, research has rarely established a connection between role-play and intrinsic motivation, and between role-play and the satisfaction of students' basic psychological need to feel related to other people (i.e., relatedness) (14). Our study investigated whether medical consultation peer role-play influences students' intrinsic motivation for medical L2 learning and the satisfaction of their needs for relatedness. With the addition of medical role-play to the curriculum, we expect to see a significant increase in students' intrinsic motivation to learn a medical L2. As peer interaction is central to role-play, we also expect to find that students' relatedness needs are satisfied. At the same time, role-playing activities are more realistic and can help students to learn better. Hence, we expect that role-play will improve students' medical L2 competence as well.

In medical consultation peer role-play, one student plays the role of doctor, whilst another student plays the role of patient. Consequently, students inevitably spend half of their time role-playing patients, although this may seem less relevant to their future careers as doctors. This raises a question: Is playing the role of a patient a valuable use of time? Earlier studies have shown that students can benefit from an observer's viewpoint, indicating that playing the role of a patient can be valuable for providing feedback (15, 16). Considering the perspective of a patient was also found to facilitate the patient care capacity of the students (17). To date, however, few studies have investigated whether role-playing as a patient can provide additional value to medical L2 learning (18). We, therefore, conducted an exploratory study in which we interviewed students after role-playing to find out if playing the part of a patient assisted them in learning a medical L2. We anticipated that playing the role of the patient would be an efficient way to learn a medical L2 and that it would offer students an additional perspective on how to improve their medical L2.

In summary, this article addressed the following research questions:

1. How does medical consultation peer role-play influence medical students' (a) intrinsic motivation and feeling of relatedness, and their (b) medical L2 competence development?
2. How does the patient role in peer role-playing affect students' medical L2 learning experience?

## Method

### Design and intervention

We used a one-group pretest–posttest design with mixed methods. Participants were 3rd-year international medical students who had registered for a course in medical Dutch and whose first language was not Dutch. Spanning 6 weeks, the course was divided into six 2-h sessions. Each session started with 1.5 h of teaching in medical Dutch, which was then followed by 0.5 h of practice in the form of peer role-play. During these half-hourly intervention sessions, students used simple role-play prompts to conduct two impromptu medical consultations in Dutch. For this activity, students were paired off randomly, with one student taking on the role of the doctor and the other playing the patient. After acting out the first medical consultation in Dutch, the students swapped roles and played the same scenario again. Hence, at the end of the course, they had role-played a total of 12 medical consultations, six of which were in the role of a doctor and six as a patient.

### Participants

A total of 19 students who had registered for the course in medical Dutch volunteered to participate in our study and signed the consent form. In total, four students did not continue with this study: three of them were granted exemptions from the course stating their Dutch language skills were sufficient and one did not participate in both the posttest and the semi-structured interview. As a result, 15 students fully participated in the study (*M*_age_ = 22.9 years, *SD* = 3.43). Each participant received a 20-euro voucher as compensation for the additional time spent on the questionnaires and interviews. We obtained ethical approval from the Ethics Review Committee of the Faculty of Health, Medicine and Life Sciences, Maastricht University (FHML-REC/2021/128).

### Materials

#### Scales measuring intrinsic motivation, feeling of relatedness, and competence

Intrinsic motivation and the feeling of relatedness and competence were considered factors that positively affected students' medical consultations as well as their medical L2 learning (19, 20). Within the framework of self-determination theory, we measured students' intrinsic motivation using the 4-item IMES subscale of the adapted academic motivation scale (13). Students' feelings of relatedness and competence were measured through the adapted need satisfaction scales—relatedness subscale (RSS) and competence subscale (CSS), each contained three items (21). Cronbach's alphas for the adapted IMES, RSS, and CSS were tested at 0.85, 0.79, and 0.75, respectively (see Appendix A). Administered at the beginning and the end of the course in medical Dutch (see Appendix A), the questionnaires were all scored on a 7-point scale.

#### Peer-assessment of students' competence in medical Dutch

To measure the extent to which the students who role-played the doctor were competent in the medical Dutch language, we used a peer-rated checklist. At the end of each role-play, the student who acted as the patient used this checklist to rate the language competence of their peer who played the doctor. The checklist measured the following four dimensions: (1) attitude and self-expression; (2) listening skills; (3) question style; and (4) structure (see Supplementary material). The assessor chose “yes” = 1 or “no” = 0 to indicate if they were satisfied with their peer's performance in each dimension, yielding a ratio of “yes” ratings that ranged from 0 to 100%. The higher this ratio, the higher the level of competence in medical Dutch for a particular dimension.

#### Final course test

At the end of the course, students underwent a final test that comprised a speaking quiz and a writing quiz. The speaking quiz consisted of another role-play in which students performed an impromptu medical consultation in Dutch for a random medical condition. In this case, the student played the role of the doctor, and the instructor played the role of the patient. After this oral examination, students were required to submit a written medical report in Dutch. Once both tests were completed, the instructor gave the students a final score between 1 (very bad) and 10 (outstanding), with a minimum score of 6 to pass the course. We used students' mean speaking and writing grades as a measure of their competence in medical Dutch.

#### Semi-structured interviews

We held semi-structured interviews with students to assess the learning environment and any changes in their medical Dutch language proficiency and consultation competence as a result of them playing the role of the patient. An example of a question we asked to explore students' experiences of this role read: “What struck you after playing the patient's role, compared to playing the doctor's role?.” Three authors of the present study (HY, MA, and JvM) jointly prepared the interview guide, which was then tested by colleagues and subsequently refined (see Supplementary material).

### Procedure

**Figure 1:**
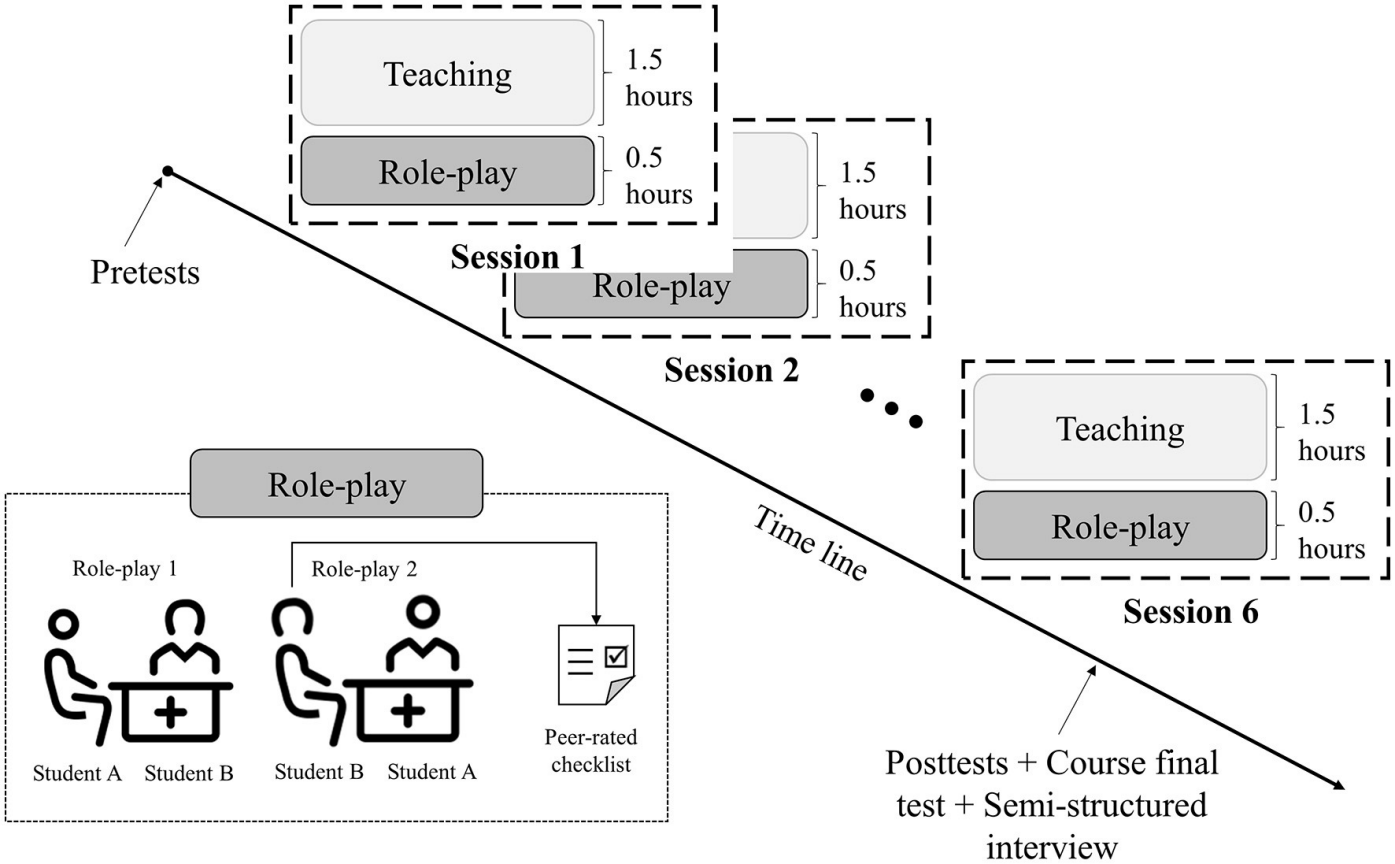
Design of the medical Dutch course combined with role-play.

We first invited students to complete the pretest questionnaires that measured their intrinsic motivation and the satisfaction of their needs for relatedness and competence at the beginning of the course (see Figure 1). In addition to the items from the IMES, RSS, and CSS scales, these questionnaires also comprised demographic questions (gender, age, and time spent learning Dutch). During the 6-week course, students attended weekly sessions in which they performed two Dutch medical consultation role-plays (one as the doctor and one as the patient) that lasted 30 min. At the end of each session, students used the peer-rated checklist to evaluate the competence of their peers. After the last session, participants completed the questionnaires again as a post-test and participated in semi-structured interviews. The interviews were followed by the final course test and we collected students' final speaking and writing grades as an indicator of their competence in medical Dutch.

### Data analysis

To provide a comprehensive overview of participants' L2 experiences, we first performed a descriptive analysis of their demographic data. We then sought to answer research question 1a, by analyzing the pre- and posttest responses to the IMES and RSS scales and performing a thematic analysis of the interview questions on intrinsic motivation. To answer research question 1b, we analyzed the pre- and posttest responses to the CSS scale, the peer-rated competence checklist data, and students' final course grades, which together triangulated their L2 competence in medical Dutch. As a final step, we analyzed the interview data to answer research question 2 and find out if and how the experience of playing the role of a patient contributed to students' development of medical Dutch.

All three scales of the pre- and post-questionnaires were analyzed using the Mann-Whitney U-test through SPSS 26.0 (IBM Corp., Armonk, USA). The data from the peer-rated competence checklists were subjected to Friedman's test. Finally, to scrutinize the semi-structured interview data, we followed Braun et al.'s (22) guidelines for thematic analysis, using ATLAS.ti 22.0 (ATLAS.ti Scientific Software Development GmbH, Berlin, Germany). Authors HY and AI first individually developed the initial coding framework. After that, the coders discussed the two resulting code sets, tested them on the second dataset, and modified them if necessary. They subsequently completed a second round of coding, after which HY discussed the outcome in separate meetings with the other researchers MA and JvM.

## Results

### Demographic information

Table 1 presents the demographic information of participants, whose nationalities were as follows: German (4), French-speaking Belgian (2), Spanish (2), Italian (2), and Dutch international (5). “Dutch international” refers to non-native Dutch speakers who had over 4 years of experience in the Dutch language before attending university. Consequently, their proficiency in Dutch corresponded to a B2 level of the Common European Framework of Reference for Languages (CEFR).

**Table 1:** Demographic information of participants.

**Demographic information**	**Participants (*n* = 15)**
Age	22.9 (± 3.4) years
**Gender**	
Female	10
Male	5
**Time spent learning Dutch**	
2–3 years	10
>4 years	5

### Medical consultation role-play improved IMES and fulfiled relatedness

**Figure 2:**
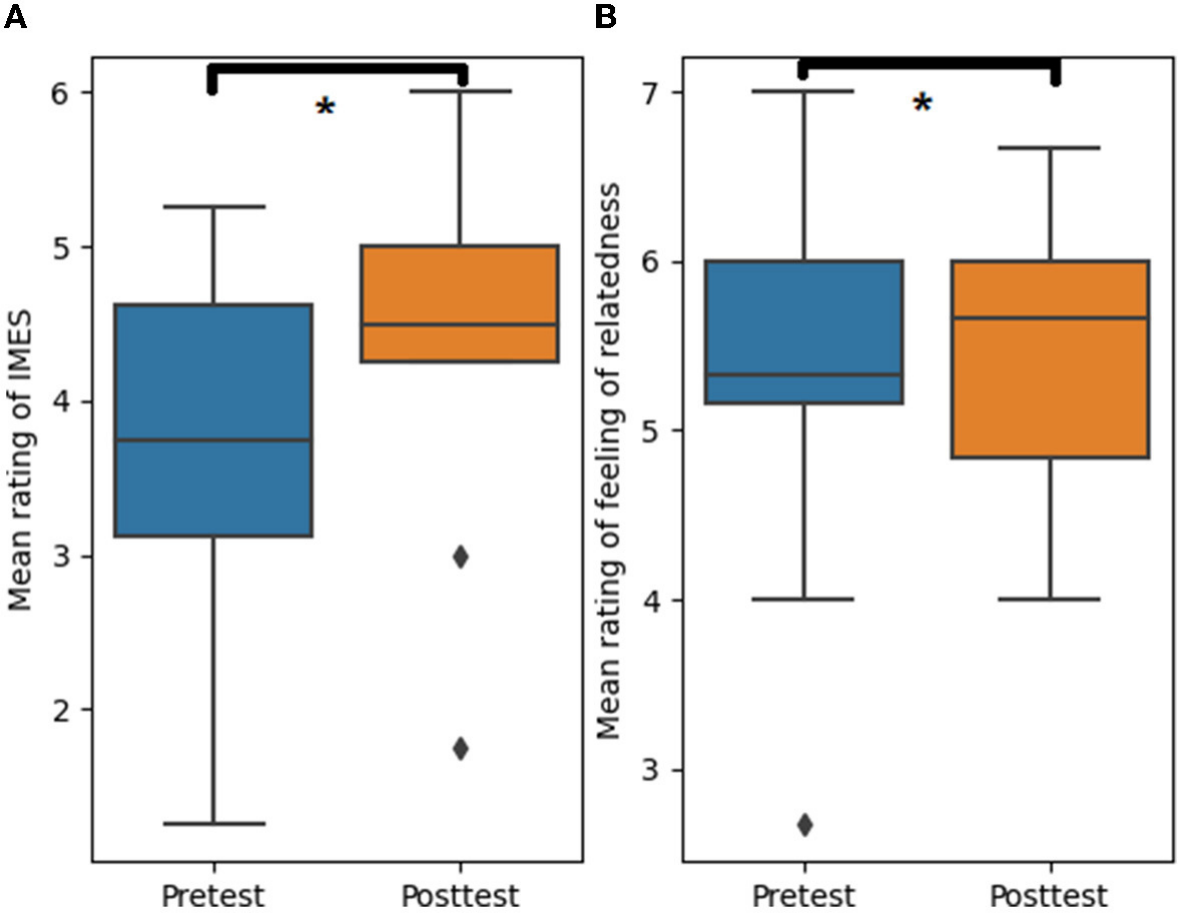
Effect of role-play in medical students' L2 learning on intrinsic motivation to experience stimulation [**(A)**, *p* = 0.02, R = 0.22] and feeling of relatedness [**(B)**, *p* = 0.041, R = 0.13]. The asterisks indicate the significant differences between the pretest and post-test (*p* < 0.05).

A comparison of pre- and posttest responses indicated that students' IMES from medical L2 learning increased from median (IQR) = 3.75 (1.75) to median (IQR) = 4.50 (0.75); *p* = 0.02, R = 0.22 (see Figure 2A). Similarly, their feeling of relatedness improved from median (IQR) = 3.33 (0.33) to median (IQR) = 3.67 (1.00); *p* = 0.041, R = 0.13 (see Figure 2B). Our analysis of the interview data further revealed two themes—motivational experience and supportive peer interaction—which suggested that role-play indeed helped improve students' IMES from medical L2 learning. In the following paragraphs, we will elucidate the said themes.

#### Supportive peer interaction

In many role-play settings, we observed that students supported and assisted each other in achieving their goals, provided feedback to one another, challenged one another's conclusions and reasoning, and explored the perspective of others. Such supportive peer interactions afforded students opportunities to satisfy their need for relatedness, which helped shape a more cooperative learning environment. The relatively high level of role-play simulation, moreover, assisted them in achieving their independent learning goals:

“I think it's quite useful because the people in our class have very different levels. Yeah, language skills, now, for sometimes you are the person with the highest skills and therefore you can more or less help the other …, but on the other hand, if you have someone who is much more experienced, then, of course, you can learn from their phrasing and just copy some of theirs.” (Participant 1)

#### Motivational experience

While observing other students' better performance in medical Dutch SPC sessions, students are inspired to strive toward the achievements of their peers which motivate them to improve.

“I don't have a clear overview in my head of what I want to say, what I want to ask. I see it, a lot of other students that they're really good at it, and that keeps me motivated to improve myself.” (Participant 13)

More importantly, the interview responses suggested that students found role-play predominantly joyful and relaxing. It allowed them to have new and exciting experiences outside of the traditional classroom setting. Moreover, students were able to apply newly acquired knowledge of the language in a simulated situation for the first time, which enhanced the learning process:

“You know, then you end up being the person in the room; you and your friend can just, like, have a bit of [fun], whilst making jokes, don't be all too super serious, and then, yeah, memorize it better, you know, but it's more fun.” (Participant 4)

### Medical consultation role-play supported medical L2 competence development

**Figure 3:**
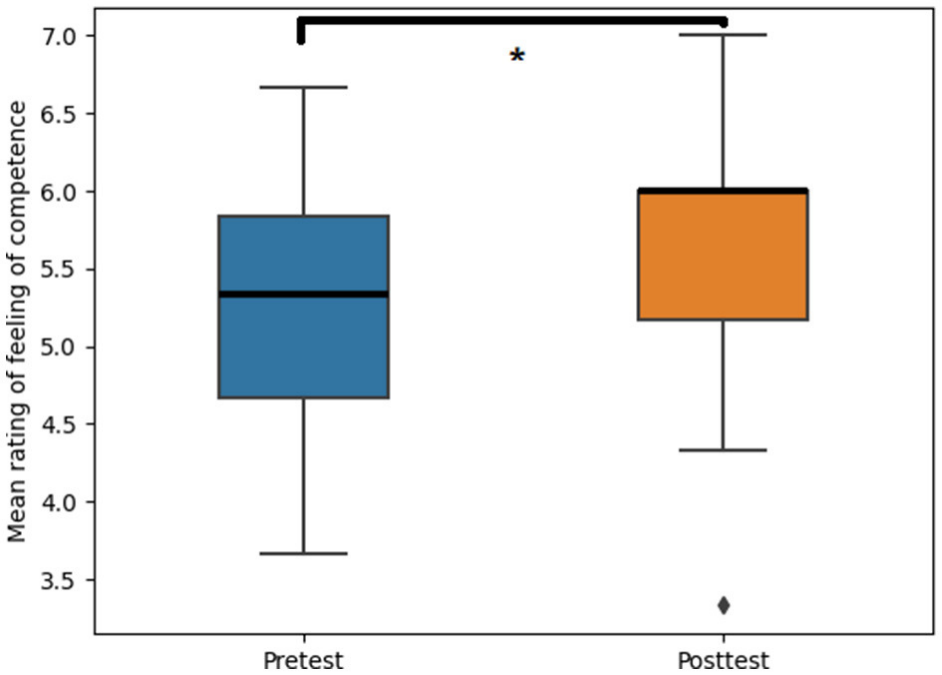
Effect of role-play in medical students' L2 learning on the feeling of competence (*p* = 0.014, R = 0.64). The asterisks indicate the significant differences between the pretest and posttest (*p* < 0.05).

**Figure 4:**
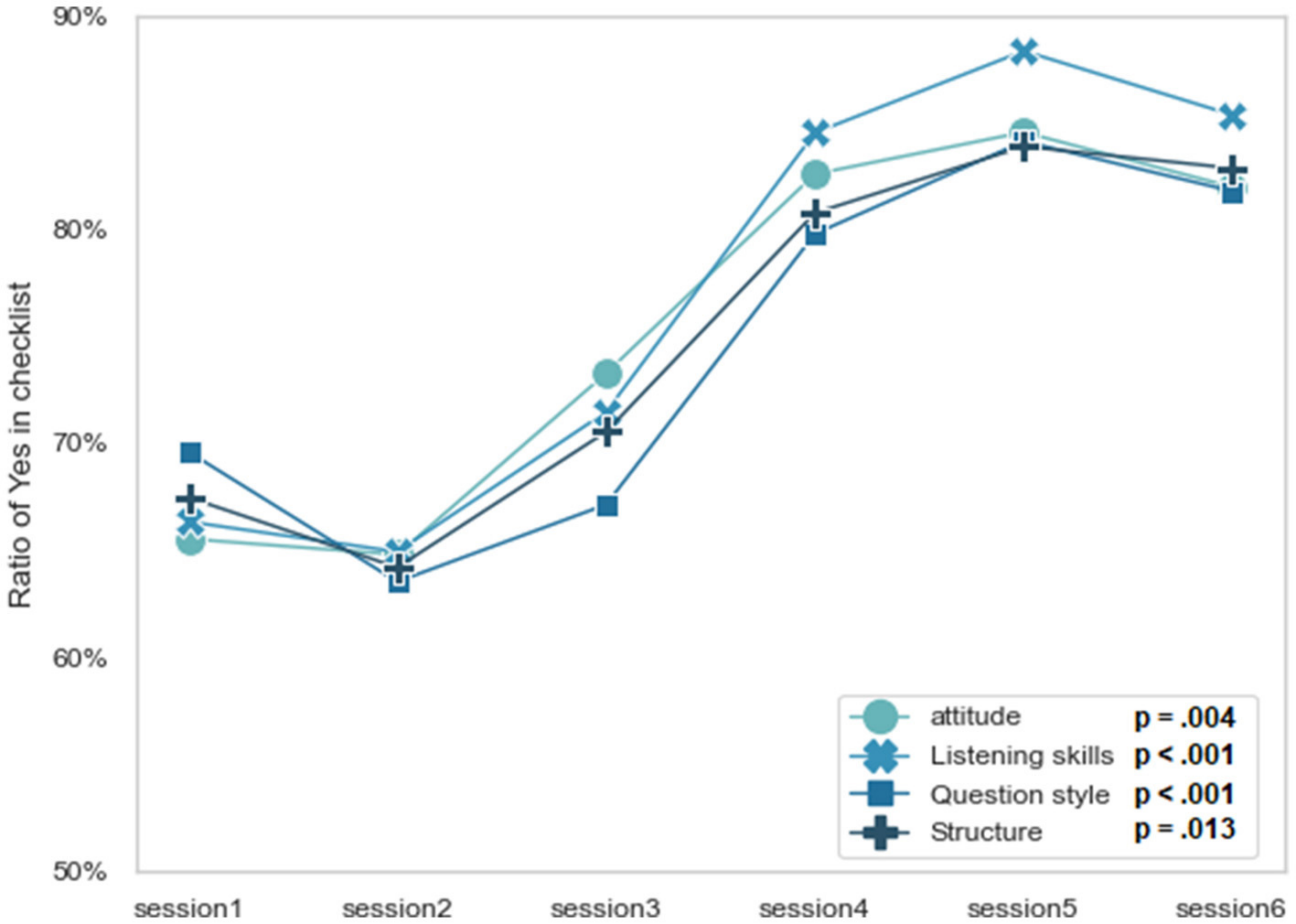
Ratio of “yes” ratings on the student–doctors' peer-rated competence checklists across six sessions. The Friedman test was conducted for each dependent variable, with significant results found for attitude [$\chi_{\left(5\right)}^{2}$ = 17.11, *p* = 0.004], listening skills [$\chi_{\left(5\right)}^{2}$ = 33.02, *p* < 0.001], question style [$\chi_{\left(5\right)}^{2}$ = 21.95, *p* < 0.001], and structure [$\chi_{\left(5\right)}^{2}$ = 14.45, *p* = 0.013].

A scrutiny of our data confirmed the assumption that role-playing facilitated medical L2 competence development. First, students reported a higher feeling of competence in the posttest [Median (IQR) = 5.67 (0.77)] compared to the pretest [Median (IQR) = 4.91 (0.71); *p* = 0.014, R = 0.64; Figure 3]. Second, ratings on the medical Dutch competence checklist also revealed growth in all four dimensions across the sessions (Figure 4). More specifically, Friedman's test showed a significant and gradual increase in the ratio of yeses on the said checklist throughout the course's six sessions [Attitude: $\chi_{\left(5\right)}^{2}$ = 17.11, *p* = 0.004, Kendall's W = 0.23; Listening skill: $\chi_{\left(5\right)}^{2}$ = 33.02, *p* < 0.001, Kendall's W = 0.44; Question style: $\chi_{\left(5\right)}^{2}$ = 21.95, *p* < 0.001, Kendall's W = 0.31; and Structure: $\chi_{\left(5\right)}^{2}$ = 14.45, *p* = 0.013, Kendall's W = 0.31]. These findings demonstrated that students' medical L2 competence improved as the course progressed. Finally, students' final course grades were relatively high, with an 8.7 (± 1.21) for the oral examination and an 8.4 (± 0.93) for the written assignment on a 10-point scale.

### Playing the patient in the role-play enhanced the medical L2 learning experience

To understand the impact of playing the patient on medical L2 learning, we subjected the interview results to additional scrutiny. We identified three more themes in addition to the ones previously specified: (1) Setting up a role-play environment for medical L2 learning; (2) utilizing the patient's role to benefit medical L2 learning; and (3) revisiting the doctor's role from a different angle. In the paragraphs to come, we will elaborate on these themes.

#### Setting up a role-play environment for medical L2 learning

The students suggested that they could be challenged more by stepping up the level of authenticity. This could be done by refining the role-play script so that students would be prompted to practice new knowledge rather than apply simpler knowledge they had already acquired (e.g., vocabulary and phrases from early teaching courses or out-of-class experiences). As one of the students mentioned:

“I think setting the role-play as realistic as possible and not to be scared to add extra information (in the instructions), because now our role-play instruction is usually only a simple paragraph.” (Participant 10)

“I do think more detailed scenarios would definitely help. And maybe just even having the scenarios in English, so the students have to do the work of translating it in their head and try to understood without it being kind of spoon-fed and you're able to read the paper, maybe just make them actively think also while being the patient.” (Participant 8)

#### Utilizing the patient role to benefit medical L2 learning

Participants noted that their experiences of playing the patient afforded them the opportunity to apply their medical L2 knowledge, which helped create a sense of flow when speaking Dutch:

“I'm pretty sure that we do learn to use some phrases that the patients would. The grammar is going better, I think. Most importantly, the flow of the language and the flow of the words is just coming more easily and you don't have to think about it.” (Participant 14)

#### A novel patient perspective on the doctor's role

Another advantage of playing the patient, according to participants, was that they could witness and learn from both how their “doctor” peers applied what they had just learned and which errors they made. Moreover, by observing their peers from a patient perspective, they gained a deeper knowledge of how to use medical L2 in the role of the doctor:

“The advantages to watch the other person is, so you get to observe them and that can also help you at simulation and learn from what they did, well, just as much as from their mistakes.” (Participant 9)

## Discussion

The first question we addressed in the present study was how medical consultation role-plays affect students' IMES and to what extent they satisfy students' need for relatedness in medical L2 learning (research question 1a). Previous researchers in the realm of traditional language learning have argued that students require high amounts of intrinsic motivation to support their learning (23). Our findings suggest that incorporating role-play enhances students' intrinsic motivation for medical L2 learning. In addition to fulfiling students' need for relatedness, the role-play activity also offered peer interaction opportunities in the classroom that benefited students' learning of a medical L2. These findings support the assertion of previous research studies that role-playing promotes peer relatedness satisfaction and the formation of peer friendships (24, 25). We think it plausible that this satisfaction was the product of students having the opportunity to gradually strengthen peer relationships over the course's 6 weeks. Yet, we do not exclude the possibility that these relationships had already been formed to a certain degree before the course, as students had already completed 2 years of study and were therefore familiar with each other. Nevertheless, we think it likely that the intervention had a reinforcing effect on the satisfaction of students' need for relatedness. We welcome future studies that validate this effect by adding a control group, for instance, 1st-year undergraduates who have not yet established such relationships.

The next question we sought to answer in this study (research question 1b) was how the role-play exercise affected students' medical L2 competence development. We did so by triangulating multidimensional data that enabled us to arrive at a logical and realistic interpretation. More specifically, we derived three types of data in the form of students' self-perceptions, peer assessments, and instructor's final course grades. The results of the pre- and post-questionnaires corroborated the peer assessment checklist data. The instructor's final course grades, in turn, objectively reflected the level of students' language competence development. We found that (1) students' fulfillment of their need for competence increased throughout the course; (2) the number of “yes” ratings on the peer-rated competence checklist increased in a similar fashion; and (3) students' final course grades were high. Based on these observations, we concluded that role-play also supported students' medical L2 competence development. Similar findings have been reported by Susan Galloway (26) who contended that incorporating role-playing into courses would foster students' competency growth. Since studies in L2 learning have inferred that intrinsic motivation and satisfaction of the need for relatedness both support students' competence development (27–32), we argue that peer role-playing is an effective strategy to improve students' medical L2 competence. Thus, we recommend that designers of medical L2 courses integrate peer role-play as a crucial component. The last question we addressed investigated the effect of playing the patient in medical consultation peer role-play on students' medical L2 learning experience (research question 2). Our thematic analysis revealed that such activity was beneficial in several ways. Whilst on the one hand playing the patient offered students an additional opportunity to practice their medical L2 expressions, on the other it allowed them to assess and improve the language abilities of their peers playing “doctor.” Although previous studies have highlighted the benefits of role-playing (33, 34), they did not home in on the benefits that may come with role-swapping and learning from a patient perspective. As such, our findings offer novel insights into medical consultation role-play, particularly from a language-learning perspective.

What also stood out in our study was that students preferred role-play scripts that were more elaborate. In their view, this would lessen the burden of their impromptu conversations, whilst guaranteeing conversation quality and preventing them from going off-topic. According to Alshammari (24), highly detailed role-plays in a pharmacy context would also make students more dependent. An unintended consequence might be that students perceive the task to be less difficult. As van der Hoorn and Killen (35) already pointed out, elaborate scripts decrease role-play authenticity, in turn lowering the challenge and, with that, learning task difficulty. A more authentic role-play, on the other hand, makes the activity more appealing to students (36). Admittedly, such open learning tasks may be difficult for students in the short term, as they require more creativity. In the long run, however, they will benefit students for they present “desirable difficulties” (35). Practically, instructors should find a balance between role-play authenticity and script refinement that best suits the intended learning outcomes.

To recap, our study found that role-play activities, by enhancing students' intrinsic motivation and satisfying their need for relatedness, aid the medical L2 learning process. As such, they also enhance students' medical L2 competence development. Furthermore, we discovered that acting as a patient in a medical Dutch role-play is an additional and beneficial approach for students to learn a medical L2. However, a few limitations need addressing. First, our sample size was small, which did not allow us to add a comparison group to control for other experimental variables, such as the presence of peer relationships. Second, we welcome future research that investigates how role-play can be designed in such a way that student dependency is reduced without compromising role-play authenticity.

## Conclusion

In this study, we have identified several benefits of medical consultation peer role-play for medical L2 learning. These include boosting students' intrinsic motivation and feeling of relatedness and contributing to the development of their medical L2 competence. In addition, we discovered that it is also valuable to have students play the role of the patient because the exercise helps to create a language-learning flow and obtain a patient perspective on how to improve medical L2. We invite future scholars to investigate the role-play setting in order to find the right balance between authenticity and a relaxed learning atmosphere. We encourage instructors to introduce role-play in medical L2 learning and to develop the meaningful role of the patient to support learning.

## Data availability statement

The original contributions presented in the study are included in the article/Supplementary material, further inquiries can be directed to the corresponding author.

## Ethics statement

The study was approved by the Ethics Review Committee of the Faculty of Health Medicine and Life Sciences, Maastricht University (FHML-REC/2021/104). The patients/participants provided their written informed consent to participate in this study.

## Author contributions

HY, MA, and JM conceived of the presented idea. MA and JM verified the analytical methods. HY and AI analyzed the data. HY drafted the manuscript under the supervision of MA, SK, and JM. All authors contributed to the article and approved the submitted version.

## Appendix

**Appendix A:** Pre- and post-questionnaire items.

The RSS and CSS scales measuring the satisfaction of students' basic psychological need for relatedness and competence (21).	IMES subscale measuring students' intrinsic motivation to experience stimulation (13).
7-point Likert scale.	7-point Likert scale.
1. In my study, I feel the people I interact with really care about me.	1. For the intense feelings I experience when I am communicating my own ideas to others.
2. In my study, I feel I am perfectly integrated into a group.	2. For the pleasure that I experience when I learn from watching others on video.
3. In my study, I feel very close and connected with other people.	3. For the pleasure that I experience when I feel completely absorbed by the learning activities.
4. In my study, I feel I am very good at the things I do.	4. For the “high” feeling that I experience while learning from various interesting course sessions.
5. In my study, I feel highly effective at what I do.	
6. In my study, I feel I can accomplish even the most difficult tasks.	
