# Supplementary material for: Second language learning role-play: effects of patient and doctor roles on motivation and competence
Source: Front Med (Lausanne). 2023 Jun 14;10:1163267. doi: 10.3389/fmed.2023.1163267 (PMC10304282; doi:10.3389/fmed.2023.1163267)
Supplement: Supplementary file 1 [file Data_Sheet_1.DOCX]

Supplementary Material

**Questionnaire items in pretest and posttest.**

Basic psychological need satisfaction scale for relatedness and competence 7-point Likert scale survey (Chen et al., 2015)

1. In my study, I feel the people I interact with really care about me.
2. In my study, I feel perfectly integrated into a group.
3. In my study, I feel very close and connected with other people.
4. In my studies, I feel I am very good at the things I do.
5. In my studies, I feel highly effective at what I do.
6. In my studies, I feel I can accomplish even the most difficult tasks.

Intrinsic motivation to experience stimulation: a 7-point Likert scale survey (Vallerand et al., 1992)

1. For the intense feelings I experience when I am communicating my own ideas to others.
2. For the pleasure that I experience when I learn from watching others on video.
3. For the pleasure that I experience when I feel completely absorbed by the learning activities.
4. For the "high" feeling that I experience while learning from various interesting course sessions.

**Peer-rated medical Dutch competence Checklist**

De checklist bestaat uit een reeks goed uitgewerkte criteria aan de hand waarvan u een uitstekende consultatie kunt doen.

U kunt snel fouten of omissies vinden door na te gaan of de opgesomde gedragingen tijdens het rollenspel en de consultatie correct werden gedemonstreerd.

Meer "Ja's" op de checklist duiden op betere prestaties van de arts (u/uw medestudent).

Beoordeel nu de prestaties van jezelf/je medeleerling aan de hand van het rollenspel/simulatie.

Let op: Als het een zelfreflectie is, dan ben jij de dokter. Als het geen zelfreflectie is, dan sturen we de feedback naar de student die de rol van de dokter heeft gespeeld, zodat hij/zij zijn/haar prestaties kan verbeteren.

Q6 Vul uw naam in.

Q8 Beoordeel je jezelf?

Q28 Vul de naam in van de persoon voor wie u een rating wilt

| **Checklist voor gesprek met de patiënt** | | | | | | |
| --- | --- | --- | --- | --- | --- | --- |
| Naam: | Wat is uw rol van rollenspel of gesimuleerde raadpleging? | | | | Datum: | |
|  | Consultant | Patiënt | Leraar | |  | |
| Afmetingen | Te observeren gedragingen | | | Ja | | Geen |
| Houding en zelfexpressie | De student sprak met correcte grammaticale en gebruikspatronen. | | |  | |  |
|  | De leerling sprak in een passend tempo. | | |  | |  |
|  | De student praat beleefd en informeel met patiënten. | | |  | |  |
|  | De student gebruikte onderwerp specifieke (niet te algemene en niet te specifieke) woordenschat om vragen te beantwoorden en uitspraken te doen. | | |  | |  |
|  | De student sprak op een gepast volume. | | |  | |  |
|  | De leerling kan praten met een goede intonatie en uitspraak. | | |  | |  |
|  | De leerling kan spreken met een goede uitspraak. | | |  | |  |
| Luistervaardigheden | De student toont begrip en empathie voor de toestand van de patiënt. | | |  | |  |
|  | De cursist kan accuraat reageren op opmerkingen van de patiënt. | | |  | |  |
|  | De student toonde belangstelling en aanmoediging voor de antwoorden van de patiënt. | | |  | |  |
|  | De student luistert naar de stem van de patiënt, het spreken, de ademhaling, het hoesten en het kreunen. | | |  | |  |
| Vraag stijl | Leerlingen gebruiken open vragen correct als ze meerdere antwoorden hebben. | | |  | |  |
|  | Deze leerling gebruikt correct de gesloten vraag voor de vragen met specifieke antwoorden. | | |  | |  |
|  | De vraag van de student is gemakkelijk te begrijpen. | | |  | |  |
|  | De vraag van de student is duidelijk en ondubbelzinnig. | | |  | |  |
|  | De leerling gaat door met het stellen van meer gedetailleerde vragen op het juiste moment / en bij de juiste gelegenheid. | | |  | |  |
|  | De informatie die de student aan de patiënt gaf was duidelijk. | | |  | |  |
| Structuur | De student heeft aan het begin van het gesprek een inleiding gehouden. | | |  | |  |
|  | De student maakte mini samenvattingen tijdens het gesprek. | | |  | |  |
|  | De student maakte samenvattingen aan het einde van het consult. | | |  | |  |
|  | De student benadrukte de overgang tussen verschillende fasen. | | |  | |  |
|  | De uitdrukking van de student is gestructureerd en samenhangend. | | |  | |  |
|  | De student heeft het hele overleg zeer goed geleid. | | |  | |  |
|  | Deze student zorgt ervoor dat de doelstellingen van elke fase van het raadplegingsproces duidelijk zijn. | | |  | |  |
|  | Deze student heeft een goede controle over de consultatietijd. | | |  | |  |

**Peer-rated medical Dutch competence Checklist English Version**

The checklist consists of a series of well-developed criteria on the basis of which you can make an excellent consultation.

You can quickly find errors or omissions by verifying that the listed behaviors were correctly demonstrated during the role play and consultation.

More "Yes" on the checklist indicates a better performance of the doctor (you/your fellow student). Now rate yourself/your fellow student's performance on the basis of the role play/simulation.

Note: If it is a self-reflection, then you are the doctor. If it is not self-reflection, then we send the feedback to the student who played the role of the doctor so that he/she can improve his/her performance.

| **Checklist for conversation with the patient** | | | | | | |
| --- | --- | --- | --- | --- | --- | --- |
| Object Name: | Who’s performance you are giving feedback to? | | | | | Date: |
|  | 🞏 Self | 🞏 Peer | 🞏 Teacher | | |  |
| Dimensions | Behaviors to Observe During Patient Consultations: | | | Yes | No | |
| Attitude and self - expression | The student spoke with correct grammar and usage patterns. | | |  |  | |
|  | The student spoke at an appropriate pace. | | |  |  | |
|  | The student talks politely and informally with patients. | | |  |  | |
|  | The student used topic specific (neither too general nor too specific) vocabulary to answer questions and make statements. | | |  |  | |
|  | The student spoke at an appropriate volume. | | |  |  | |
|  | The student can speak with good intonation and pronunciation. | | |  |  | |
|  | The student can speak with good pronunciation. | | |  |  | |
| Listening skills | The student shows understanding and empathy for the patient's condition. | | |  |  | |
|  | The student can accurately respond to patient comments. | | |  |  | |
|  | The student showed interest and encouragement in the patient's responses. | | |  |  | |
|  | The student listens to the patient's voice, speaking, breathing, coughing and moaning. | | |  |  | |
| Question style | Students correctly use open-ended questions when they have multiple answers. | | |  |  | |
|  | This student correctly uses the closed question for the questions with specific answers. | | |  |  | |
|  | The student's question is easy to understand. | | |  |  | |
|  | The student's question is clear and unambiguous. | | |  |  | |
|  | The student continues to ask questions in more detail at the appropriate time / and opportunity. | | |  |  | |
|  | The information the student provided to the patient was clear. | | |  |  | |
| Structure | The student made an introduction at the beginning of the conversation. | | |  |  | |
|  | The student made mini summaries during the conversation. | | |  |  | |
|  | The student made summaries at the end of the consultation. | | |  |  | |
|  | The student emphasized the transition between different phases. | | |  |  | |
|  | The student's expression is structured and coherent. | | |  |  | |
|  | The student presided over the whole consultation process very well. | | |  |  | |
|  | This student ensures that the goals of each stage of the consultation process are clear. | | |  |  | |
|  | This student has good control over the consultation time. | | |  |  | |

**Semi-structured interview guidelines**

Name of interviewee:

Name of interviewer:

Date:

**Establishing Rapport**

How are you going? How do you enjoy the course?

**Experience of the role of the patient**

1. How do the role plays and the simulated patient consultations work on your course?
   1. How many times did you play the role of patient during the role plays?
   2. How many different partners did you have when playing the patient role?
   3. How do you evaluate the use of different partners during the role play?
   4. How do you evaluate how your patient is acting? (Do you consider yourself a good patient actor or not?)
   5. What surprised you only after you played the patient role?

- How do you evaluate this patient experience?
  1. How did it influence your consultation initiation? (hint: establishing rapport, **identifying reasons**)
  2. How has it influenced your information gathering? (hint: patient problem **exploration**, understanding the patient's **perspective**)
  3. How has it influenced your consultation structure? (hint: summarizes, signposting, logical sequencing, timing)
  4. How has it influenced your relationship-building? (hint: **non-verbal behavior**: eye contact, vocal cue, facial expression...; note-taking, demonstrating confidence; developing**rapport**: being not judgmental, using empathy, dealing with embarrassing and disturbing topics or physical examination or  pain; sharing**thinking**, explaining rationale)
  5. How has it influenced explanation and planning? (Hint: share the decision-making process with the patient.)
  6. How has it influenced your closing session? (hint: summarize and final check?)

**Learned from playing the role of patient**

Now that you have played the role of a patient and have firsthand experience of all the feelings that a patient would go through while being treated by a doctor,

- Would you consider making any changes in your behavior as a doctor to be more accommodating for your patients? If yes, what would you change?

**Influence on professional behavior**

- As you were playing the role of patient every week, could you explain the series of mental actions and the changes you considered making to your professional behavior as a doctor as a result of playing the role of patient?
  1. Establish professional therapeutic relationships with patients and their families.
  2. Elicit and synthesize accurate and relevant information, incorporating the perspectives of patients and their families.
  3. Share health care information and plans with patients and their families.
  4. Engage patients and their families in developing plans that reflect the patient’s health care needs and goals.
  5. Document and share written information about the medical encounter to optimize clinical decision-making, patient safety, confidentiality, and privacy.

**Language learning**

- What are the effects of roleplaying patients on your medical Dutch learning?

**Evaluation of playing the role of patient**

- Could you please again specify the advantages and disadvantages of you playing the role of patient and the effects it had on your behavior as a doctor in the simulated consultations?
- What will you say to next year’s students when you try to encourage them to be willing to take on the role of patient during the role plays? What are the effects you would be talking about?
